# Supplementary material for: Characterization of brain dystrophins absence and impact in dystrophin-deficient Dmdmdx rat model
Source: PLoS One. 2020 Mar 11;15(3):e0230083. doi: 10.1371/journal.pone.0230083 (PMC7065776; doi:10.1371/journal.pone.0230083)
Supplement: S1 Raw images — (PDF) [file pone.0230083.s001.pdf]

WT

For figure 5A conception

Dp71

Primary antibody : Dys2, 1/100
Secondary antibody: Dako P0447 1/5000
Exposure time : 1 min

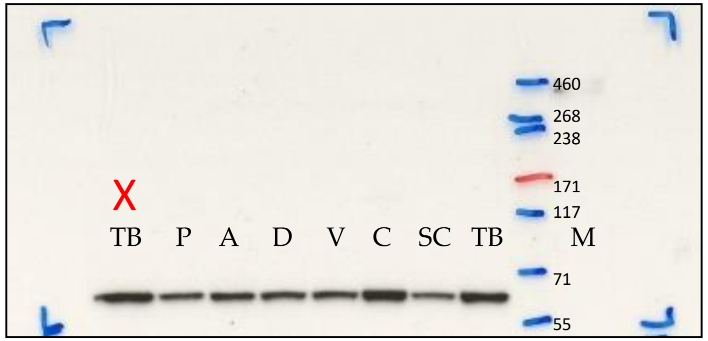

GAPDH

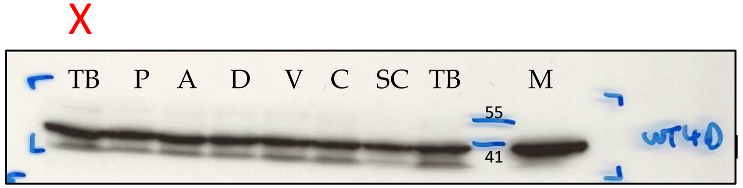

Primary antibody : GAPDH 1/10000
Secondary antibody: Dako P0449 1/2000
Exposure time : 1 min

Dp140

Primary antibody : Dys2, 1/100
Secondary antibody: Dako P0447 1/5000
Exposure time : 30 min

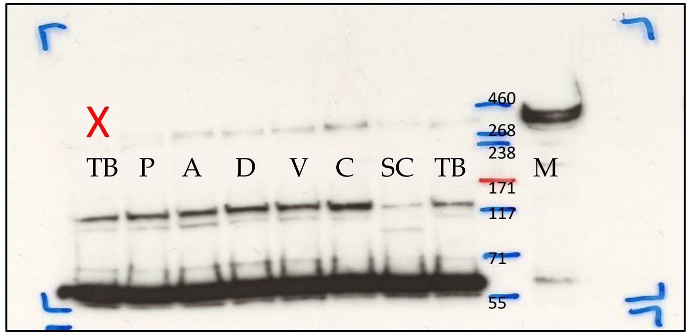

GAPDH

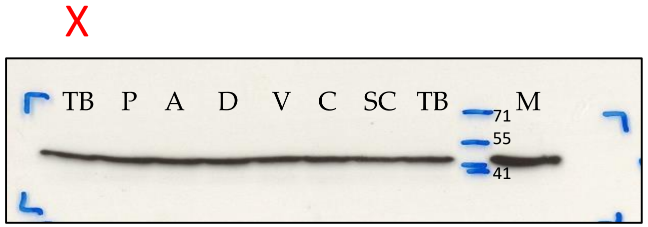

Primary antibody : GAPDH 1/10000
Secondary antibody: Dako P0449 1/2000
Exposure time : 5 min

Dp427

Primary antibody : MANEX 1011C, 1/250
Secondary antibody: Dako P0447 1/5000
Exposure time : 3 h

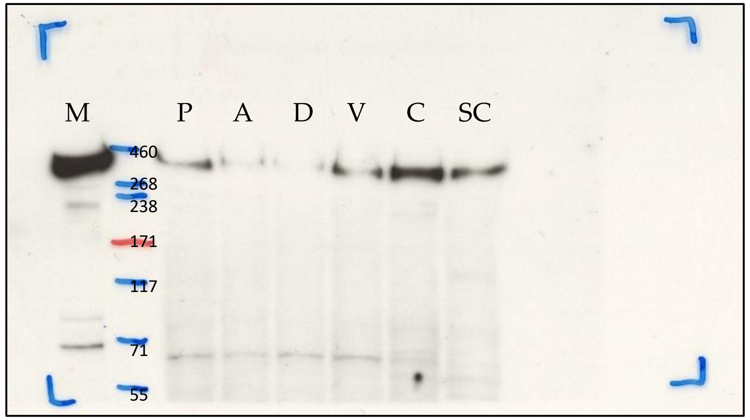

GAPDH

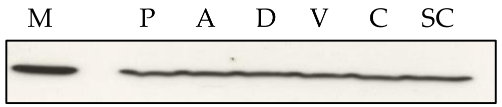

Primary antibody : GAPDH 1/10000
Secondary antibody: Dako P0449 1/2000
Exposure time : 5 min

Dmd^mdx

For figure 5A conception

Dp71

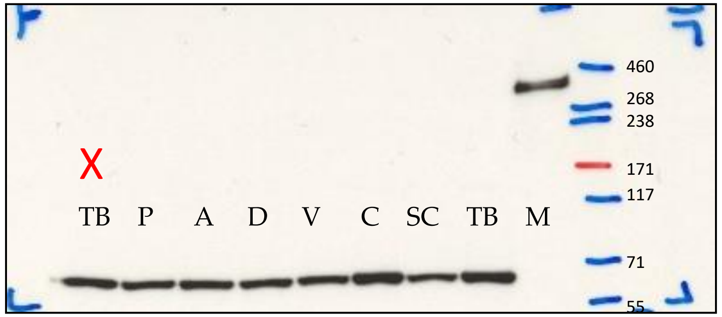

Primary antibody : Dys2, 1/100
Secondary antibody: Dako P0447 1/5000
Exposure time : 1 min

GAPDH

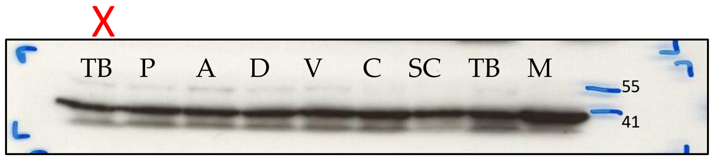

Primary antibody : GAPDH 1/10000
Secondary antibody: Dako P0449 1/2000
Exposure time : 1 min

Dp140

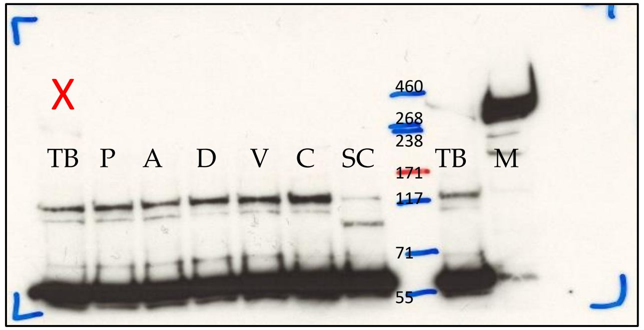

Primary antibody : Dys2, 1/100
Secondary antibody: Dako P0447 1/5000
Exposure time : 30 min

GAPDH

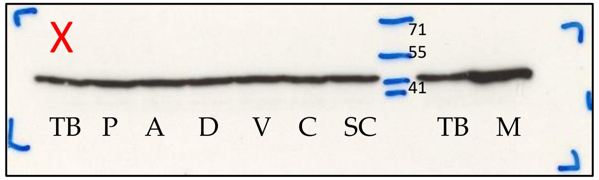

Primary antibody : GAPDH 1/10000
Secondary antibody: Dako P0449 1/2000
Exposure time : 5 min

Dp427

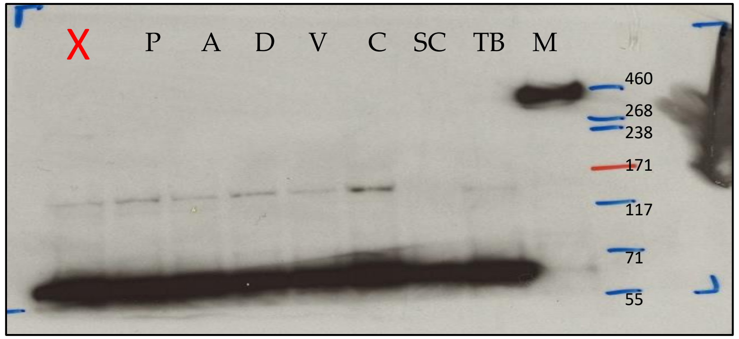

Primary antibody : MANEX 1011C, 1/250
Secondary antibody: Dako P0447 1/5000
Exposure time : 3 h

GAPDH

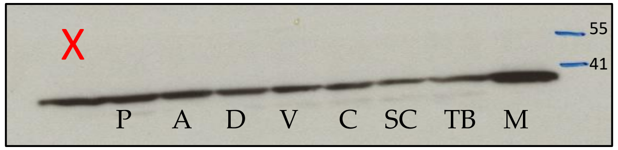

Primary antibody : GAPDH 1/10000
Secondary antibody: Dako P0449 1/2000
Exposure time : 5 min
